# Who Is an “Orthodontist?”

**Published:** 1915-06

**Authors:** Martin Dewey


### WHO IS AN "ORTHODONTIST?".

BY MARTIN DEWEY, D.D.S., M.D.

At this time when the profession is more or less divided as to whether they are practicing "orthodontia" or "orthodontics," the question has been raised as to who is an "orthodontist?" What are the moral and legal obligations assumed by one who advertises to the public by word or sign that he is an orthodontist? The position of the orthodontist from a legal standpoint has never been clearly determined.

In speaking of the legal standing of an orthodontist, the practice of orthodontia has in the past been controlled by the same laws as have governed the practice of dentistry, and it is well and proper that it should. As a result any one qualified to practice dentistry could also practice orthodontia. In fact, orthodontia is today included in the course of instruction in most of our leading dental colleges, and the legal conditions are such that any one who has fulfilled the requirements to practice dentistry can also practice orthodontia; but having the legal right to practice dentistry and orthodontia does not make one an "orthodontist." You may then ask: What is an orthodontist?

From the accepted use of the word, an orthodontist is one who treats malocclusion of the teeth and facial deformities. He is supposed to possess more than the ordinary degree of skill. At the present time, we have no way of determining when a person possesses the required skill and training necessary to make him an orthodontist. There are requirements which one must fulfill to practice dentistry or medicine, and if the requirements are not met, legal difficulties are encountered. Since the practice of orthodontia is really a highly specialized branch of dentistry, and since all practitioners of orthodontia are in a sense specialists, the only legal difference existing between the dentist and the orthodontist is the same as exists between the physician and the surgeon. A surgeon must be a physician but a physician need not necessarily be a surgeon. We understand a surgeon to be one who possesses more than ordinary skill along certain lines. Rhinologists and pathologists are physicians who possess more than the ordinary skill and judgment in their respective branches of medicine. Therefore a medical practitioner who advertises by word, card, or sign that he is a surgeon, rhinologist or pathologist must necessarily bring to the aid of the one employing him as such, both in diagnosis and treatment, that degree of skill and knowledge which is ordinarily possessed by those who devote special study and attention to that particular branch of medicine. The general public is led to believe that any one designating himself by any term that denotes a specialty of medicine or dentistry is qualified to do work along that particular line far better than could be done by the average practitioner. This is a well known fact in medical jurisprudence and covers the field of the orthodontist just as positively.

There is no law which prevents any one legally engaged in the practice of dentistry from treating malocclusion, but there are moral and legal reasons why he should not call himself an orthodontist.

The moral reason is that the public has learned that the term ''orthodontist'' denotes one skilled above the ordinary in the treatment of malocclusion. This skill can be obtained in three ways:

First, by long years of practice, during which period one devotes a certain amount of time to the treatment of malocclusion and proves by the finished results that he has attained more than ordinary skill. Second, by years of association with someone who has attained extraordinary skill and thereby becomes familiar with methods that will enable him to be an orthodontist in every sense of the word. Third, by a course of study and training in some school which has proven its ability to so educate students that they can treat malocclusion with more than the ordinary skill.

Any one who has had the proper training as mentioned in the above paragraph, would certainly be qualified to be known as an orthodontist. However, we find a number of men who by sign, card or word are claiming to be orthodontists, when in reality they do not possess the necessary qualifications. Any one who claims to be an orthodontist and begins the treatment of a case of malocclusion, with the patient or the parents of the patient believing he possesses more than the ordinary skill, is legally and morally responsible. If he does not show more than the ordinary amount of skill in the treatment of the malocclusion, he is liable. If suit was begun, and it was found that the person claiming to be an orthodontist could not qualify according to one of the three above mentioned requirements, judgment could be easily obtained.

We have known of several cases where practitioners, though not possessing the qualifications mentioned, have placed signs on their doors, or inserted notices in the papers, claiming to be orthodontists. There is no legal way to prevent any one entitled to practice dentistry from calling himself an orthodontist; but to do so, makes one liable in the same manner that a specialist of medicine is liable. Therefore, the orthodontist assumes a greater responsibility than the general practitioner when he treats malocclusion. The orthodontist is required by law to use greater skill and obtain better results. In assuming these extra responsibilities, the orthodontist is entitled to fees which will reimburse him for the responsibilities assumed and the skill used in the treatment of the case. Unfortunately, it is the increased fees and not the increased responsibility which has led many men to call themselves "orthodontists." About the fees and the responsibility, we will have more to say later.—*International Journal of Orthodontia.*

